# Nature-based interventions for people living with dementia and their carers: a scoping review

**DOI:** 10.3389/frdem.2026.1818223

**Published:** 2026-08-24

**Authors:** Manpreet K. Gill, Shareefa Thekkan, Danine Irwin

**Affiliations:** Dementia Adventure, Chelmsford, United Kingdom

**Keywords:** carers, dementia, gardening, green care farms, green space, horticulture, nature-based interventions, outdoor activity

## Abstract

**Introduction:**

Nature-based interventions are increasingly recognised as a promising non-pharmacological approach to support people living with dementia and their carers. Although a growing body of research has explored the relationship between nature engagement and dementia, the evidence remains dispersed across a wide range of intervention types, settings and outcome measures. This review aimed to identify and map the available evidence relating to nature-based interventions for people living with dementia and their carers.

**Methods:**

A scoping review was conducted following the Arksey and O’Malley framework and reported in accordance with PRISMA-ScR guidelines. Searches were undertaken across PubMed, Scopus, Web of Science, Emerald Insight and the Cochrane Library. Evidence selection was conducted independently by two reviewers. Data were charted and synthesised using narrative synthesis and thematic categorisation.

**Results:**

A total of 108 studies were included. Six overarching themes were identified: Therapeutic Gardens and Environmental Design (*n* = 33), Horticultural and Gardening Interventions (*n* = 30), Green Care Farms and Nature-Based Services (*n* = 22), Nature Exposure, Access and Participation (*n* = 16), Technology-Based Nature Interventions (*n* = 5), and Development and Implementation of Nature-Based Services (*n* = 2). Across intervention types, engagement with nature was consistently associated with positive outcomes relating to wellbeing, social participation and meaningful activity. Findings also highlighted the importance of accessibility, environmental design and opportunities for continued participation in everyday life.

**Discussion:**

The evidence suggests that nature-based interventions may offer benefits beyond wellbeing alone, supporting identity, social participation and meaningful engagement for people living with dementia and their carers. However, the evidence base remains unevenly developed, with limited research relating to implementation, blue spaces, technology-based approaches and underrepresented populations. Future research should focus on improving accessibility, understanding implementation and broadening the range of nature-based approaches available to people affected by dementia.

## 1 Introduction

Dementia is a collective term for a group of progressive neurodegenerative conditions that may affect memory, cognition, senses and everyday functioning. Globally, more than 57 million people are living with dementia, with prevalence expected to rise substantially over coming decades as populations age (GBD 2019 Dementia Forecasting Collaborators, 2022). In the United Kingdom, approximately 1 million people are currently living with dementia, a figure projected to exceed 1.4 million by 2040 (Wittenberg et al., 2020). As dementia is both progressive and incurable, there is increasing emphasis on supporting people to live well with the condition through approaches that promote wellbeing, independence, participation and quality of life.

Dementia can have wide-ranging impacts on both people living with the condition and their carers, affecting independence, confidence, mood and social participation, whilst creating emotional, physical and practical challenges. Consequently, increasing attention has been directed toward non-pharmacological, person-centred approaches (Luxton et al., 2026). Activities aligned with an individual’s preferences, abilities and life experiences are considered particularly valuable in supporting identity and continued participation in everyday life (Tierney et al., 2023).

Amongst the range of non-pharmacological approaches available, nature-based interventions have received increasing attention within dementia research and practise. This interest is underpinned by a broader evidence base linking engagement with natural environments to improved physical, psychological and social wellbeing (Barragan-Jason et al., 2023; Bryer et al., 2024; Twohig-Bennett and Jones, 2018). The importance of nature connectedness and meaningful engagement with natural environments as potential pathways through which these benefits occur has also been reported (Richardson et al., 2021). Previous reviews examining nature-based approaches in dementia care have reported positive outcomes relating to wellbeing, engagement, social participation and quality of life (Collins et al., 2023; Mmako et al., 2020; Ramos et al., 2026; Whear et al., 2014). Additionally, reviews have explored specific aspects of nature-based dementia care, including the effectiveness of nature-based interventions for reducing agitation (Choe et al., 2025) and the role of gardens and outdoor spaces for people living with dementia in their own homes and communities (Bennett et al., 2022; Newton et al., 2021).

Despite this growing body of evidence, existing reviews have largely focused on specific intervention types, settings or populations. Reviews have examined topics such as horticultural therapy, therapeutic gardens, outdoor activities and environmental design, but a broader synthesis encompassing the full range of nature-based interventions remains limited.

This scoping review was therefore conducted to identify and map the available evidence relating to nature-based interventions for people living with dementia and their carers. Specifically, the review sought to examine reported benefits and outcomes, and identify gaps within the existing evidence base to inform future research and practise. The review addressed the following research question: ‘How does exposure to nature or nature-based interventions impact or benefit people living with dementia and their carers?’

## 2 Methods

### 2.1 Scoping review design

The review was guided by the Arksey and O’Malley (2005) framework, with reporting structured according to the PRISMA-ScR (Preferred Reporting Items for Systematic Reviews and Meta-Analyses extension for Scoping Reviews) guidelines.

An internal review protocol was developed to guide the conduct of the review. This protocol was not registered in a public repository.

### 2.2 Information sources and search strategy

A comprehensive search was conducted from April–May 2026 across five electronic databases: PubMed, Scopus, Web of Science, Emerald Insight, and the Cochrane Library. The search strategy combined dementia-related terms with nature-based intervention and environmental exposure terms. Search terms were adapted to the requirements of each database, using the Boolean operators AND OR to combine terms consistently across searches.

The search strategy was developed iteratively following preliminary scoping of the literature and identification of relevant keywords. Initial testing indicated substantial variation in the terminology used to describe nature-based interventions and environmental exposures within the dementia literature. Consequently, a series of complementary searches was undertaken to maximise sensitivity and reduce the risk of omitting relevant studies.

All searches combined dementia-related terms with nature-related concepts. The dementia search block consisted of:

(dementia OR Alzheimer*)

Three thematic search domains were then applied:

Search A: Green and blue space.

(dementia OR Alzheimer*) AND (“green space” OR greenspace OR “blue space” OR “green care”)

Search B: Gardens and horticulture.

(dementia OR Alzheimer*) AND (garden* OR horticultur* OR “horticultural therapy” OR “sensory garden*” OR “therapeutic garden*” OR “community garden*”)

Search C: Nature-based approaches.

(dementia OR Alzheimer*) AND (“nature-based” OR “nature based” OR “nature therap*” OR ecotherap* OR “eco-therapy” OR “forest bathing”)

All searches were conducted separately for each database, and for each search the exact search string, date of search, and number of records retrieved were documented to ensure reproducibility.

Search results for each database are provided in Appendix 1.

### 2.3 Eligibility criteria

Articles were screened against predefined inclusion and exclusion criteria.

Inclusion criteria:

- Peer-reviewed empirical studies (quantitative, qualitative, mixed-methods)
- Focus on people living with dementia and/or carers
- Examination of nature-based, green space, blue space, or outdoor environmental exposure or interventions
- Published in English between 2000 and 2026

Exclusion criteria:

- Studies not specific to dementia populations
- Dementia prevention-only studies without intervention or lived experience focus
- Interventions without a central nature-based component
- Reviews, protocol papers, conference abstracts, posters, and unpublished literature
- Non-English publications

### 2.4 Evidence selection

All records identified through database searching were imported into Rayyan, where duplicates were automatically removed.

Evidence selection was conducted in two stages:

1. Title and abstract screening
2. Full-text screening

Both stages were conducted independently by two reviewers, with disagreements resolved through discussion and consensus.

Reasons for exclusion at full-text stage were recorded.

### 2.5 Data charting

A structured data charting form was used to extract relevant information from included sources of evidence. Extracted data included:

- Author(s) and year
- Country of study
- Study sample size/demographic
- Aim of study
- Key findings relevant to dementia and/or carers

### 2.6 Evidence synthesis

A narrative synthesis approach was used to map and summarise the evidence.

Findings were grouped into higher-order themes based on similarities in intervention characteristics, settings and reported outcomes. Themes were refined iteratively through repeated review of the extracted data until a coherent thematic framework was developed.

No formal critical appraisal of included sources was undertaken, consistent with scoping review methodology.

## 3 Results

### 3.1 Study selection

**Figure 1:**
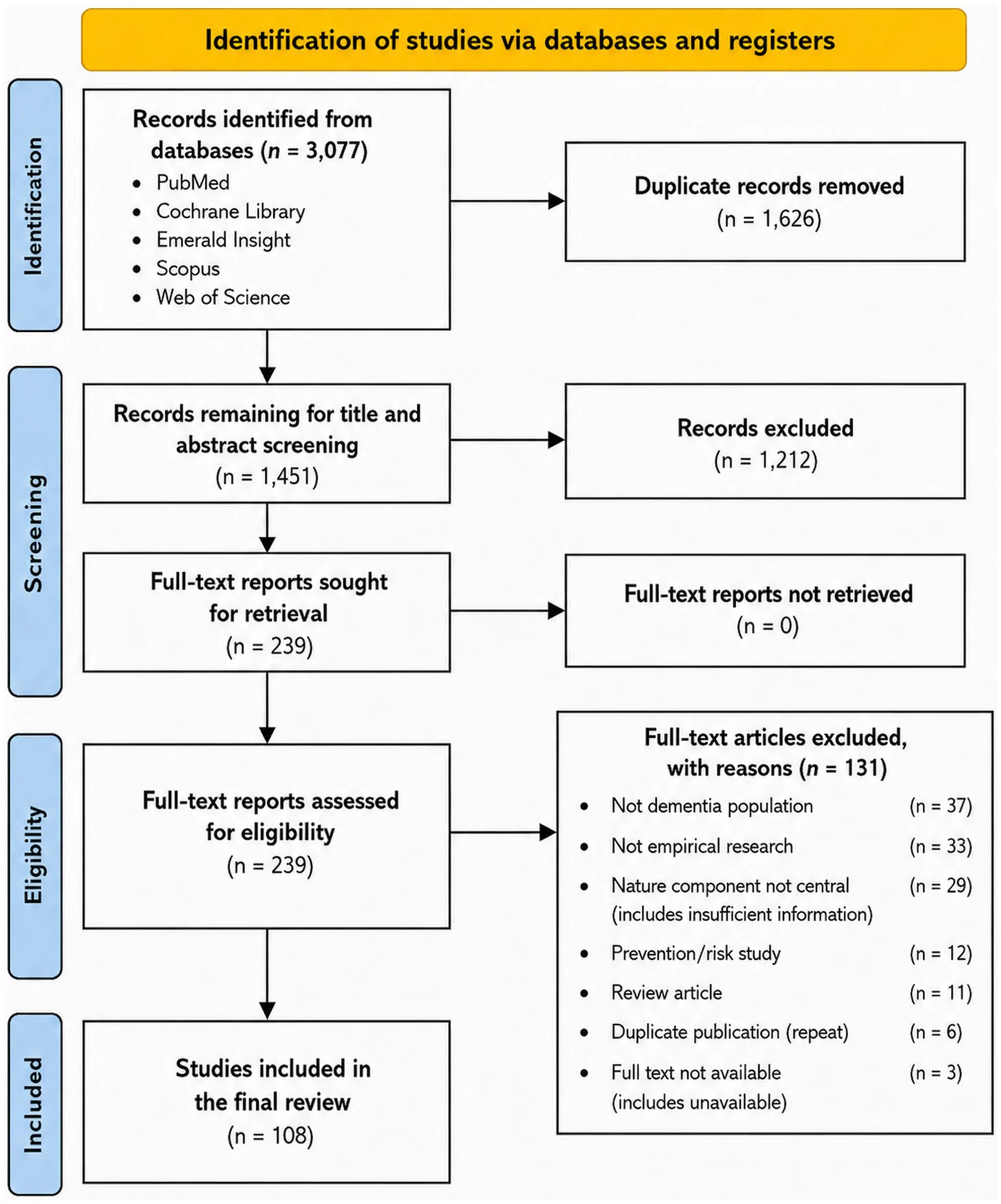
PRISMA flow diagram of study selection.

The database search identified 3,077 records. Following removal of 1,626 duplicate records, 1,451 records remained for title and abstract screening against the eligibility criteria. Of these, 1,212 records were excluded, leaving 239 articles for full-text review. Following full-text assessment, 131 articles were excluded for not meeting the inclusion criteria. A total of 108 studies were included in the final review. The study selection process is presented in the PRISMA flow diagram (Figure 1).

### 3.2 Characteristics of included studies

A total of 108 studies published between 2000 and 2026 were included in the review. The studies were conducted across 21 countries, with the largest number originating from the United Kingdom (*n* = 21), the Netherlands (*n* = 20), and the United States (*n* = 13) (Table 1). Participants included people living with dementia, family carers, care staff, healthcare professionals, volunteers and service providers across a variety of settings, including residential care, community environments, hospitals, adult day services, therapeutic gardens and Green Care Farms.

**Table 1:** Geographic distribution of included studies by country (*n* = 108).

Country	*n*
United Kingdom	21
Netherlands	20
United States	13
Australia	9
South Korea	7
Other countries (*n* = 16 countries)	38
Total	108

Six overarching themes were identified through thematic categorisation of the included studies (Table 2). Therapeutic Gardens and Environmental Design (*n* = 33) and Horticultural and Gardening Interventions (*n* = 30) represented the largest areas of evidence, followed by Green Care Farms and Nature-Based Services (*n* = 22). Nature Exposure, Access and Participation accounted for *n* = 16 studies, whilst Technology-Based Nature Interventions (*n* = 5) and Development and Implementation of Nature-Based Services (*n* = 2) represented smaller areas of evidence.

**Table 2:** Distribution of included studies across the six overarching themes identified through thematic categorisation (*n* = 108).

Overarching theme	*n*
Therapeutic gardens and environmental design	33
Horticultural and gardening interventions	30
Green care farms and nature-based services	22
Nature exposure, access and participation	16
Technology-based nature interventions	5
Development and implementation of nature-based services	2
Total	108

The findings relating to each theme are presented in Sections 3.3–3.8.

### 3.3 Therapeutic gardens and environmental design

Therapeutic Gardens and Environmental Design represented the largest area of evidence identified within the review (*n* = 33). Terminology varied across the included studies, and some terms overlapped in meaning. For clarity, a therapeutic garden is used here as a broad term for garden environments intentionally designed or used to support health, wellbeing or participation. More specific labels used by individual studies included healing, sensory, wander, enriched, Japanese and indoor gardens. The evidence also examined the design and evaluation of dementia-friendly outdoor spaces in residential care, hospital and community settings.

Therapeutic garden use was associated with improvements in mood, wellbeing, quality of life, engagement and social interaction (Akindejoye et al., 2025; Cox et al., 2004; Detweiler et al., 2008; Edwards et al., 2013; Lai et al., 2023; Liao et al., 2020; Meneghetti et al., 2023; Motealleh et al., 2022; Van der Velde-van Buuringen et al., 2026). One notable study found that improvements in patient mood were observed after relatively brief exposure to nature, with no further measurable gains reported beyond 80–90 min (White et al., 2018).

Reductions in agitation, apathy, depression and other psychological symptoms were also reported with garden use (Akindejoye et al., 2025; Detweiler et al., 2008; Edwards et al., 2013; Jevtic et al., 2025; Lai et al., 2023; Meneghetti et al., 2023; Motealleh et al., 2022; Murphy et al., 2010; Pedrinolla et al., 2019).

Therapeutic gardens were associated with physical, cognitive, physiological and social outcomes. A wander garden – a secure outdoor walking space – was linked to fewer and less severe falls and reduced medication use for residents of a dementia care facility (Detweiler et al., 2009). An enriched garden, comprising an outdoor sensory garden supplemented by 12 purpose-designed modules targeting cognition, independence, walking and balance, improved cognition, activities of daily living, balance and mobility amongst nursing home residents (Bourdon and Belmin, 2021). Exposure to a purpose-designed indoor therapeutic garden, used as an environmental intervention within a residential care setting, reduced medication use, cortisol levels and blood pressure (Pedrinolla et al., 2019). Japanese-style gardens were primarily used as viewing environments, with participants observing the gardens whilst seated rather than engaging in walking or gardening activities. Viewing these gardens reduced heart rate and physiological stress and supported visual engagement, verbalisation and memory retrieval (Goto et al., 2017; Goto et al., 2014; Goto et al., 2018). Further outcomes of garden views included changes in nervous system activity and emotional wellbeing, improved self-consciousness and body representation, and lower agitation (Gueib et al., 2020; Koura and Ikeda, 2016; Qian et al., 2026). Therapeutic gardens also created opportunities for communication, identity, autonomy and social connection through meaningful relationships, positive risk-taking and active citizenship (Fielder and Marsh, 2021; Marsh et al., 2018). Garden-based art therapy supported independence, self-expression and psychological safety (Clarke et al., 2025), whilst garden redevelopment and natural, cultural and multisensory features provided stimulation and prompted emotional, cognitive and social responses valued by residents, carers and visitors (Cox et al., 2004; Ng et al., 2023; Yzoard et al., 2017). Garden use also reduced depression in one study without improving cognition (Jevtic et al., 2025).

Accessibility, usability and integration into everyday care influenced garden use, notably in residential and hospital care. Rivasseau Jonveaux et al. (2013) described the development and evaluation of a hospital-based healing garden designed around neuropsychological and artistic principles for people living with dementia. All 14 patients valued the garden’s accessibility, views and opportunities for walking, whilst 50% reported a calming effect, 33% improved mood and 41.6% improved communication (Rivasseau Jonveaux et al., 2013). Accessible design, sensory features, social spaces, meaningful activities and responsiveness to changing needs were also identified as important features of dementia-friendly gardens (Giebel et al., 2022; Oher et al., 2024). However, in other studies the differences between design intentions and actual use were reported, and outdoor spaces were not always fully integrated into care (De Boer et al., 2018; Heath, 2004; Hernandez, 2007). Personal, institutional and resource-related barriers were also found to restrict access (Fielder and Marsh, 2021).

Findings relating to sustained use were mixed. In one study, daily garden use was feasible within residential care and was perceived to benefit residents, relatives and staff (van der Velde-van Buuringen et al., 2021). However, in a similar study, low garden use was reported in a residential care facility despite positive feedback and reductions in neuropsychiatric symptoms and falls amongst residents (Lai et al., 2023). Van der Velde-van Buuringen et al. (2026) evaluated a multicomponent intervention that included improvements to the garden and access routes, alongside staff, family and organisational support for everyday garden use. The intervention temporarily increased garden use and social activity, although garden use returned to baseline at follow-up and improvements in quality of life were limited to social relationships (van der Velde-van Buuringen et al., 2026).

### 3.4 Horticultural and gardening interventions

Horticultural and gardening interventions formed a substantial component of the evidence base (*n* = 30). In this review, horticultural therapy refers to structured or facilitated programmes using plants and gardening-related activities to support therapeutic goals and wellbeing. The evidence included community gardening initiatives, gardening activities within residential care and adult day services, home gardening, and interventions involving family carers. Interventions varied considerably in duration, intensity, setting and participant characteristics, reflecting the diverse ways in which gardening and horticulture have been used to support people affected by dementia.

Horticultural and gardening interventions were associated with a range of positive psychosocial outcomes. The most frequently reported benefits related to wellbeing and mood (*n* = 15), engagement and participation (*n* = 12), cognitive outcomes (*n* = 11) and social connection (*n* = 10). Gardening activities supported productive engagement, positive mood and meaningful participation, whilst enabling participants to experience a sense of achievement, connect with others and maintain links with hobbies and interests (Borella et al., 2023; Gigliotti and Jarrott, 2005; Hall et al., 2018; Hewitt et al., 2013; Jarrott and Gigliotti, 2010; Jarrott et al., 2002; Kim et al., 2020; Morris et al., 2021; Smith-Carrier et al., 2021; Styck and George, 2022; Vitman-Schorr et al., 2024).

An important feature of these interventions was their emphasis on active participation rather than passive exposure to nature. Activities such as planting, watering, harvesting, cooking and maintaining gardens enabled participants to contribute to tangible tasks and engage in meaningful occupation. Outcomes relating to identity, purpose, agency and self-expression were reported in eight studies. Gardening supported continuity and self-worth, created opportunities to contribute to other people and the wider community, and enabled expressions of autonomy, citizenship and empowerment (Buse et al., 2024; Hewitt et al., 2013; Noone and Jenkins, 2018; Pachana et al., 2023; Rushton et al., 2025; Smith-Carrier et al., 2021; Styck and George, 2022; Swift et al., 2024). A conceptual paper similarly proposed that horticultural therapy could support physical and psychological wellbeing and social integration amongst people living with dementia in community settings (Noone et al., 2017).

Several studies highlighted the potential for horticultural activities to support cognitive engagement through practical tasks, learning, reminiscence and sensory stimulation. Indoor gardening was associated with improvements in cognition (Lee and Kim, 2008). In a separate study, a tabletop planting game involving progressively more complex flower-combination tasks improved cognitive performance amongst people with mild or moderate dementia (Tseng et al., 2020). An indoor horticultural therapy programme also improved frontal lobe cognitive functioning (Kim and Yun, 2024), whilst outdoor horticultural activities were associated with improvements in cognition (Borella et al., 2023). Outdoor gardening also produced greater prefrontal cortex activation than indoor cognitive training (Kang et al., 2025). Improvements in cognition for women with mild cognitive impairment or mild dementia were reported following programmes of different frequencies and durations, although a shorter and more intensive programme produced greater improvements (Kim et al., 2020).

Findings were not uniformly positive. One study found no significant overall reduction in agitation and reported different responses according to participants’ levels of cognitive functioning. Participants with lower cognitive functioning appeared to experience reduced agitation, whilst those with higher cognitive functioning showed increased agitation following the intervention (Luk et al., 2011). Yang et al. (2022) reported reductions in apathy and improvements in cognition, but no observed effects on quality of life or functional capacity (Yang et al., 2022).

Opportunities for physical activity and functional participation were also identified. Studies examined weekly horticultural activity programmes, outdoor gardening sessions and horticultural therapy-based activities compared with traditional activities. Reported outcomes included activities of daily living, participation, cooperation and engagement (Jarrott and Gigliotti, 2010; Park et al., 2008; Thelander et al., 2008). However, physical and functional outcomes were less frequently examined than psychosocial and cognitive outcomes, and improvements were not consistently demonstrated.

Family carer outcomes were also reported. Gardening participation was associated with significantly lower symptoms of depression, anxiety and stress amongst family carers (Ainamani et al., 2021). Structured horticultural therapy improved carer quality of life and showed potential to reduce carer burden (Kim et al., 2020), whilst improvements in psychological wellbeing and caregiving self-efficacy were reported in a separate study (Rozani and Vitman-Schorr, 2025). Improvements in peer support and shared wellbeing were also reported when carers participated in horticultural activities alongside people living with dementia, as well as reduced distress (Borella et al., 2023; Morris et al., 2021).

Participation and responses to interventions varied according to participants’ cognitive and physical abilities. Adapted or flexible delivery supported participation across different activity types, whilst agitation outcomes differed according to cognitive functioning (Jarrott and Gigliotti, 2004; Luk et al., 2011; Thelander et al., 2008). A study of stakeholder preferences indicated that programmes delivered weekly, lasting no more than 60 min and comprising no more than 10 sessions were preferred, with psycho-emotional outcomes prioritised as the main programme goal (Lee et al., 2024).

### 3.5 Green care farms and nature-based services

Twenty-two studies examined Green Care Farms and other nature-based care services. These studies explored a range of nature-based models, including Green Care Farms, farm-based day services, social farms and outdoor care programmes that integrated activity, care and engagement with the natural environment into everyday life. Whereas horticultural interventions generally centred on participation in specific gardening-related activities, Green Care Farms and related services integrated nature into the wider organisation of care and everyday life.

Green Care Farms and nature-based services consistently supported wellbeing, social participation and meaningful engagement in everyday life. Participants were actively involved in everyday tasks including gardening, walking, food preparation and farm maintenance, enabling them to maintain valued roles, contribute to daily life and participate in activities that reflected their preferences and abilities (De Boer et al., 2017a; De Boer et al., 2017b; De Bruin et al., 2009; De Bruin et al., 2015; Ibsen et al., 2018; Rosteius et al., 2022; Rosteius et al., 2025a; Rosteius et al., 2025b; Ura et al., 2021). Compared with more traditional care settings, residents spent more time outdoors, participated in more domestic and outdoor activities, and demonstrated higher levels of physical activity, social interaction and activity engagement, alongside improvements in quality of life, wellbeing and mood (Ellingsen-Dalskau et al., 2021; Frissen et al., 2025).

The Vicenza Farm Project described a structured farm-based therapeutic horticulture programme involving 31 participants in its first year, including people with young-onset dementia. The authors reported that the model was feasible to deliver across four farms and provided opportunities for meaningful participation, purpose, identity and social connection (Dalla Costa et al., 2026). Additionally, one study reported improved dietary intake amongst attendees of a Green Care Farm compared with regular day care (De Bruin et al., 2010). Carers also reported positive experiences, including reassurance regarding care quality, greater satisfaction with care provision and appreciation of person-centred approaches (de Boer et al., 2019; De Bruin et al., 2021; Smit et al., 2022). However, one longitudinal study found no significant differences between Green Care Farm and regular day care in maintaining functional performance over time (de Bruin et al., 2012).

Implementation studies suggested that the successful delivery of Green Care Farms and nature-based services depended on organisational culture, staffing, funding, access to green space and wider policy contexts. Although several authors concluded that key characteristics of Green Care Farms and social farms could be transferred to other care settings, achieving this would require substantial organisational and environmental change (Bartlett et al., 2025; Buist et al., 2018; Hassink et al., 2019; Innes et al., 2025; Steinmann et al., 2025).

### 3.6 Nature exposure, access and participation

Sixteen studies examined how people living with dementia and their carers accessed, experienced and participated in natural environments. Unlike the previous themes, which focused on specific interventions or models of care, these studies explored the role of nature within everyday life, including walking outdoors, visiting parks and woodlands, spending time in local green and blue spaces, engaging in leisure activities and maintaining connections with meaningful outdoor places.

Access to nature was consistently described as an important aspect of everyday wellbeing rather than a formal therapeutic intervention in many studies. Natural environments supported wellbeing, mood, quality of life and enjoyment, whilst also providing opportunities for relaxation, reflection, leisure participation and continued engagement with valued activities (Connell et al., 2007; Evans et al., 2022; Evans et al., 2019; Kolster et al., 2025; Owen et al., 2024; Toubol et al., 2025; Wu et al., 2021). In the largest study, most participants with and without dementia living in assisted living facilities considered nature to be important, many wished for greater contact with nature and 83% expressed interest in participating in nature-based interventions (Kolster et al., 2025). A study involving 107 dyads found that forest bathing in urban green spaces was acceptable and feasible and was associated with reductions in carer burden, depressive symptoms and anxiety (Choy and Ma, 2025).

Several studies highlighted the contribution of natural environments to social participation, identity and connection with meaningful places. Participants described opportunities for conversation, companionship and shared experiences with family members and communities, whilst nature-based activities supported self-worth, identity, capability and continuity with previous lifestyles (Bartlett and Brannelly, 2019; Gibson et al., 2007; Mapes, 2017; Olsson et al., 2013; Owen et al., 2024; Toubol et al., 2025).

A recurring finding across the literature was the gap between the desire to spend time in nature and the ability to do so. Studies consistently identified barriers relating to accessibility, transport, safety, confidence, environmental design and risk management, alongside the importance of supportive environments and appropriate adaptations to facilitate participation (Evans et al., 2022; Nouri and Chaudhury, 2025; Page et al., 2025; Stapley et al., 2024; Van Schaik et al., 2008). Although people living with dementia often wished to maintain their participation in outdoor activities and connection with nature, practical and environmental barriers frequently restricted opportunities for participation (Bartlett and Brannelly, 2019; Stapley et al., 2024).

### 3.7 Technology-based nature interventions

Five studies examined technology-based nature interventions for people living with dementia, including virtual reality (Kim et al., 2023; Latorre et al., 2025), digital nature experiences (Kor et al., 2025), nature imagery (Eggert et al., 2015) and video-based approaches (Rados et al., 2021).

Three studies relating to technology-based nature interventions were associated with positive emotional outcomes, including improvements in engagement, alertness, mood and wellbeing (Eggert et al., 2015; Kim et al., 2023; Kor et al., 2025). Reductions in agitation and symptoms associated with sundowning were identified in one study where participants viewed a 6-min nature video (Rados et al., 2021), and reduced heart rate was recorded in participants who were exposed to nature-based virtual reality (Latorre et al., 2025). The studies highlighted the potential for technology to increase access to nature for individuals who experienced barriers to outdoor participation and reported good acceptability of technology-mediated approaches.

### 3.8 Development and implementation of nature-based services

Two studies focused on the development and implementation of nature-based dementia services (Bray et al., 2022; Hendriks et al., 2016). Rather than evaluating specific interventions, these studies explored the organisational, practical and contextual factors that influence the delivery of nature-based interventions for people living with dementia and their carers.

Key findings highlighted the importance of organisational culture, staff knowledge and skills, partnership working, access to appropriate outdoor environments, and sustainable funding arrangements. Both studies emphasised that successful implementation requires nature-based approaches to be embedded within wider systems of care and support, rather than delivered as isolated activities (Bray et al., 2022; Hendriks et al., 2016).

## 4 Discussion

This scoping review synthesised 108 studies examining nature-based interventions for people living with dementia and their carers. The findings demonstrate a substantial and growing evidence base encompassing a diverse range of nature-based approaches and environmental interventions for people affected by dementia. Across these approaches, engagement with nature was consistently associated with positive outcomes relating to wellbeing, social participation, meaningful activity, engagement and quality of life.

An important observation arising from this review is that the benefits associated with nature extend beyond wellbeing alone. Across multiple intervention types, opportunities for participation, contribution and engagement in everyday life were evident. Activities such as, but not limited to, gardening, walking, spending time outdoors and indoor interventions, enabled people living with dementia to maintain valued roles, remain connected to previous interests and continue participating in meaningful activities. These findings align with person-centred approaches to dementia care (Kitwood and Bredin, 1992) which emphasise strengths, capabilities and social participation. Nature-based interventions may therefore offer more than recreational opportunities, providing meaningful ways for people living with dementia to remain connected to their communities, interests and identities.

Whilst positive outcomes were reported across almost all themes identified within this review, the findings suggest that access to nature may be as important as the specific intervention itself. Several studies highlighted barriers relating to transport, accessibility, confidence, safety concerns and the availability of dementia-friendly environments. This suggests that one challenge is ensuring that people living with dementia can access and participate in nature-based opportunities, rather than simply increasing the availability of opportunities.

The distribution of studies included within this review provides further insight into the development of the evidence base. Research has predominantly focused on therapeutic gardens, horticultural interventions and organised programmes delivered within residential care settings and other formal services. Whilst these approaches provide valuable evidence regarding effectiveness, they may not fully reflect how people living with dementia engage with nature in everyday life. Comparatively less attention has been directed toward informal nature engagement, spontaneous outdoor participation and community-based opportunities beyond formal care services. This is particularly important given the increasing emphasis on supporting people to live well with dementia within their own homes and communities.

A further observation is that the evidence base remains unevenly developed. Although substantial evidence was identified for therapeutic gardens and horticultural interventions, relatively few studies examined technology-based interventions or development and implementation of services. Similarly, there was limited evidence specifically addressing the experiences of people living with young-onset dementia. Rare dementias were also largely absent from the included literature. The review also highlighted a notable absence of research examining blue spaces, such as coastal, river, lake and aquatic environments. Whilst these findings may reflect emerging areas of research, they also highlight opportunities for future investigation.

The findings of this review suggest that the primary challenge is no longer establishing whether nature-based approaches can benefit people living with dementia and their carers, but understanding how such opportunities can be made accessible, sustainable and available to those who wish to participate. Future research should therefore place greater emphasis on implementation, accessibility and community participation, whilst continuing to explore underrepresented approaches and populations. Such work may help ensure that the benefits associated with nature are available to a broader range of people affected by dementia.

## 5 Strengths and limitations

This scoping review provides a broad overview of nature-based interventions and environmental approaches for people living with dementia and their carers. By searching five databases and including a wide range of intervention types, settings and populations, the review was able to identify common themes across the evidence base whilst also highlighting areas where evidence remains limited.

The included studies varied considerably in terms of intervention type, setting, participant characteristics and outcome measures, making direct comparison between studies challenging. The review was also restricted to studies published in English and may therefore have excluded relevant evidence published in other languages. In addition, some areas of the evidence base were represented by only a small number of studies, limiting the conclusions that can be drawn regarding emerging nature-based approaches such as technology-based interventions and implementation research.

## 6 Conclusion

Evidence examining nature-based interventions for people living with dementia and their carers has grown substantially over the past two decades. Across a diverse range of intervention types and settings, engagement with nature was consistently associated with positive outcomes relating to wellbeing, social participation, meaningful activity and quality of life.

The findings suggest that the value of nature extends beyond wellbeing alone, supporting opportunities for participation, identity, connection and engagement in everyday life. Access to nature should therefore not be viewed solely as a recreational opportunity, but as an important component of living with dementia. The review also highlights the importance of accessibility, as benefits can only be realised when people living with dementia are able to access and engage with natural environments.

Whilst the evidence base continues to expand, important gaps remain. Future research should place greater emphasis on implementation, accessibility, blue spaces and underrepresented populations. Addressing these gaps will support the development of accessible and sustainable nature-based opportunities for people living with dementia and their carers.
