# Supplementary material for: Nature-based interventions for people living with dementia and their carers: a scoping review
Source: Front Dement. 2026 Aug 24;5:1818223. doi: 10.3389/frdem.2026.1818223 (PMC13548805; doi:10.3389/frdem.2026.1818223)
Supplement: Supplementary file 1 [file Supplementary_file_1.docx]

**9.0 Appendix 1 - Full Search Results by Database**

Core Dementia Search Terms

(dementia OR Alzheimer*)

Search A: Green and Blue Space

(dementia OR Alzheimer*) AND ("green space" OR greenspace OR "blue space" OR "green care")

Search B: Gardens and Horticulture

(dementia OR Alzheimer*) AND (garden* OR horticultur* OR "horticultural therapy" OR "sensory garden*" OR "therapeutic garden*" OR "community garden*")

Search C: Nature-Based Approaches

(dementia OR Alzheimer*) AND ("nature-based" OR "nature based" OR "nature therap*" OR ecotherap* OR "eco-therapy" OR "forest bathing")

Search Results by Database

| **Database** | **Search** | **Theme** | **Records Retrieved** |
| --- | --- | --- | --- |
| PubMed | A | Green & Blue Space | 106 |
| PubMed | B | Gardens & Horticulture | 822 |
| PubMed | C | Nature-Based Approaches | 45 |
| Cochrane Library | A | Green & Blue Space | 2 |
| Cochrane Library | B | Gardens & Horticulture | 36 |
| Cochrane Library | C | Nature-Based Approaches | 1 |
| Emerald Insight | Dementia and Garden | Green & Blue Space | 370 |
| Emerald Insight | Dementia and Horticulture | Gardens & Horticulture | 34 |
| Emerald Insight | Dementia and Outdoor | Nature-Based Approaches | 282 |
| Scopus | A | Green & Blue Space | 139 |
| Scopus | B | Gardens & Horticulture | 472 |
| Scopus | C | Nature-Based Approaches | 70 |
| Web of Science | A | Green & Blue Space | 187 |
| Web of Science | B | Gardens & Horticulture | 444 |
| Web of Science | C | Nature-Based Approaches | 67 |
| Total records identified through database searching | | | 3077 |
